# Development of a breast cancer case management information platform (BC-CMIP) module based on patient-perceived value

**DOI:** 10.3389/fonc.2022.1034171

**Published:** 2022-11-28

**Authors:** Yinyin Liang, Yan Gao, Guobing Yin, Wenjun Chen, Xiuni Gan

**Affiliations:** ^1^ Department of Breast and Thyroid Surgery, Second Affiliated Hospital of Chongqing Medical University, Chongqing, China; ^2^ Nursing Department, Second Affiliated Hospital, Chongqing Medical University, Chongqing, China

**Keywords:** patient-perceived value, breast cancer, case management, informatization, telemedicine

## Abstract

**Objective:**

To construct a content module for a breast cancer case management information platform (BC-CMIP) based on patient-perceived value (PPV).

**Methods:**

A questionnaire was used to investigate the service needs of breast cancer patients and their families for the information platform. Based on the value dimensions of PPV, the module content of the BC-CMIP was initially constructed, and the Delphi method was used to justify and revise the module content. Excel 2019 and SPSS 26.0 were used for statistical analysis.

**Results:**

The information platform includes the patient side and the medical side. The index content includes four primary indicators: functional value, emotional value, efficiency value and social value; it can realize all patient case management needs, such as diagnosis and treatment services, health education, telemedicine, treatment tracking, psychological support, case assessment and positive warning.

**Conclusion:**

Based on the PPV, the module design of the BC-CMIP is reasonable and comprehensive, and it can scientifically and effectively meet the health needs of patients and provide a theoretical basis for subsequent platform development and application.

## 1 Introduction

Breast cancer (BC) is one of the most common malignant tumours in women, ranking first in incidence. Statistics released by the World Health Organization (WHO) in 2018 showed that 2.09 million new cases of BC were diagnosed, accounting for 11.6% of the total number of new cancer cases worldwide (18 million) (1). In China, approximately 250,000 women develop BC, and approximately 60,000 die from it yearly (2). Although BC is the most common cancer among women, it is the sixth most common cause of cancer death among women and has a relatively good prognosis compared to other cancers, with a 5-year observed survival rate of 72.7%, making it a cancer with a high survival rate (3, 4). However, due to the heterogeneity of BC, the treatment protocol requires rationalized therapy in individual cases according to the characterization and stage of the disease; thus, the treatment is complex and requires a long follow-up period (5, 6). Patients face many difficulties during treatment, care and recovery, for example, stress during treatment, emotional needs, and need for knowledge about the disease (7–9). Therefore, there is an urgent need to establish an individualised model for the management of BC patients throughout the course of their care.

Currently, various management models are also being explored for BC patients. For example, the self-management model based on empowerment theory can benefit postoperative chemotherapy patients in the process of physical and psychological recovery and improve their quality of life; the model has facilitative effects and practical significance in enhancing psychological resilience, psychological adjustment, disease awareness and self-management ability (10). The multidisciplinary management model has a good effect on chemotherapy-induced nausea and vomiting among BC patients (11). The 5S health education management model is used in the health education of patients with BC (12). However, these models currently focus on a single function and fail to create a full continuum of patient care from prehospital to posthospital.

The case management model meets the need for versatility. Case management (CM) is a system of assessment, planning, service delivery, coordination and monitoring of health care for a particular condition aimed at providing and coordinating care for a specific group of patients (13, 14). In 1985, the New England Medical Center in Boston was the first to implement a nursing care system with nurses as case managers in response to a prospective payment system, and the CM model has since been applied to acute care and long-term care systems (15). In Taiwan, in response to the implementation of universal health care, CM was established for patients in 2005 (16), with significant success, especially in oncology case care (17).At present, CM is more widely used in diseases with a long course, complex treatment and high medical costs, such as patients with severe mental illness (18), dementia (19) and cancer (20). CM is an extension of in-hospital care that integrates traditional fragmented health care systems to ensure that patients receive continuous and complete care that is high in quality and efficiency (21). A case manager, who is trained in CM, is responsible for coordinating with physicians, the health care team and the patient to develop a treatment plan and goals and to ensure that the patient completes the required tests and treatments on schedule to achieve the desired goals within a predetermined time frame. Case managers can be physicians or nursing staff but are primarily nurses (22). In countries such as the USA, Australia and Taiwan, clinical practice has proven that CM led by case managers is a successful model (23, 24). Studies (25) have also found that applying a CM model to BC patients can help them return to work as soon as possible and that case managers can play an active role in screening programmes for breast and cervical cancer (26).

Convenience, comfortable environment, and faster assessment related to the treatment surroundings could foster more relaxed emotions, accompanied by patient-perceived fairness and efficiency (27, 28). Delays in appointments has increased tension and conflict (29). Patients’ dissatisfaction led many Chinese hospitals to adopt IT systems to improve convenience and workflow efficiency for patients. These e-programs in hospitals, such as electronic registration machines, electronic health record (EHR) systems, electronic payment machines, and online appointment systems, are becoming widely used in an effort to reduce the time it takes to receive medical treatment. However, the current mHealth platform for breast cancer patients does not enable management of the entire process from prehospital to posthospital.

Patient-perceived value (PPV) is an extension of customer-perceived value in the healthcare sector (30). The concept of customer-perceived value is the overall assessment of the effectiveness of a product or service when the customer’s perceived benefit is weighed against the cost to the customer. The introduction of mobile healthcare has promoted the study of PPV. Hu Rong et al. (31) proposed four dimensions of PPV as functional, emotional, social and efficiency values in the context of mobile healthcare. PPV is a better indicator of the effectiveness of healthcare services than indicators such as patient satisfaction and service experience (30).

Following an extensive literature search, we first constructed a questionnaire on patient and family needs and evaluated the information platform services, applied the PPV theoretical framework, arranged the needs in order from prehospital to posthospital, and constructed the BC-CMIP modules. After two rounds of Delphi expert consultation, the contents of the modules for constructing the BC-CMIP were finally determined, providing a theoretical basis for the subsequent construction and evaluation of the platform. This study aims to i) investigate the demand and evaluation of BC and their families for CM service programs; ii) build a BC-CMIP module based on the PPV, the demand survey results and two rounds of Delphi expert validation; and iii) construct a preliminary operational framework for the BC-CMIP.

## 2 Methods

### 2.1 Establishment of expert discussion groups

The expert discussion group consisted of 10 experts in clinical nursing management, including 3 masters, 2 masters in progress and 5 undergraduates; 1 chief nurse, 1 chief physician, 1 deputy chief nurse and 7 nurses in charge, mainly engaged in the specialist direction of BC treatment and care, nursing management and nursing education. The discussion group was responsible for developing the demand questionnaire and distributing it, extensive literature collection to develop the correspondence questionnaire, selecting the correspondence experts, statistically analysing the importance ratings of the correspondence experts for each indicator and collating the experts’ comments and suggestions, revising the strategy according to the revision principles and providing feedback to the experts.

### 2.2 Construction of the content framework

#### 2.2.1 Literature search

The literature search was conducted using a combination of subject terms and free words. PubMed, Web of Science, Embase, OVID, Cochrane Library, the Australian JBI Centre for Evidence-Based Health Care website, the US National Guideline Clearinghouse (NGC) website, and the UK National Institute for Health and Clinical Excellence (NICE) website were searched for studies published from database inception to April 1, 2022. The search strategy was built on the application of Boolean logic operators to the following keywords: (((Breast Neoplasms) OR (Breast Cancer)) OR (Mammary Cancer)) AND (((((((Mobile Applications) OR (Mobile healthmobile)) OR (Telemedicine)) OR (Telehealth)) OR (Mobile Health)) OR (Information flat)) OR (Information platform)). Using the PPV as a framework, information relevant to this study was extracted from the four dimensions of patient functional value, efficiency value, emotional value and social value, and a questionnaire on the needs of the BC-CMIP was constructed and distributed to BC patients who met the requirements.

#### 2.2.2 Survey of demand for full case management services based on perceived value theory

Prior to the distribution of the questionnaire, the expert discussion group reviewed and discussed the format and the content of the statements in the first draft of the questionnaire (**Appendix 1**). From 25 April to 30 May 2022, questionnaires were distributed to patients diagnosed with BC and their family members in a tertiary hospital in Chongqing through the online survey tool “Questionnaire Star” (an online crowdsourcing platform in China), and the purpose of the survey was first explained to them. After obtaining informed consent, they were invited to respond *via* microscan to understand the patients’ perceptions of the content and evaluation of the CM of BC patients. Inclusion criteria: (i) age ≥ 18 years; (ii) patients diagnosed with BC; (iii) family member who most often cares for the patient (limit one family member per patient); and (iv) voluntary participation in this survey. Data analysis and collation: The four dimensions of functional value, efficiency value, emotional value and social value of patients, each stage was divided into levels according to prehospital, in-hospital and posthospital, and patients and their families were asked to evaluate the specific functions of the information platform with the help of the BC case manager.

### 2.3 Correspondence method

The Delphi method is a qualitative research approach used to gain consensus through expert opinion on a real-world problem (32). The process aims to structure information on a topic about which little is known; the research questions can be answered by a panel of geographically diverse experts (32). Researchers using this method are able to obtain accurate and reliable data through multiple rounds of queries (33). The Delphi method is an appropriate choice when the research question requires gathering subjective information from experts and those working in the field (34), either to set priorities or to reach consensus where none existed before (33).

#### 2.3.1 Criteria for the selection of experts

Inclusion criteria for correspondence experts: i) long-term engagement in BC management, treatment and care; ii) high academic level in BC and CM, with outstanding research ability; iii) intermediate level or above; iv) bachelor’s degree or more; and v) voluntary participation in the consultation.

#### 2.3.2 Method of correspondence

Letters of enquiry were sent to experts by letter or email in June-July 2022 due to study site constraints. Experts rated the importance of each indicator on a 5-point Likert scale as very important, relatively important, generally important, not very important and very unimportant, assigning a score of 5, 4, 3, 2 and 1, respectively (35), and made comments, suggested changes in the revision comments column, and added new indicators (**Appendix 2**). Experts were also asked to complete a questionnaire on basic information, familiarity and basis of judgement. The degree of familiarity is divided into very familiar, familiar, generally familiar, unfamiliar and unfamiliar according to the experts’ knowledge of the issue, with values of 1.0, 0.8, 0.6, 0.4 and 0.2, respectively (36), and the basis of judgement is mainly theoretical analysis, practical experience, domestic and international references and subjective judgement. A table quantifying the basis for assigning points and their level of impact is provided in **Appendix 3**.

Principles for revision of indicators: The following cases shall be evaluated and validated by the expert discussion group to decide whether to retain, add, delete or revise the indicators, including indicators with mean importance score x< 4 or coefficient of variation CV≥25% (36), indicators proposed by experts for addition or deletion, and indicators proposed by experts for comments and suggestions. After each round of the Delphi, responses for each item are summarized and fed back.

within the subsequent questionnaire, enabling participants to consider the views of others before rerating (**Appendix 4**).

### 2.4 Statistical analysis

Data were exported in an Excel file (Microsoft Corp., Redmond, WA, USA) and analysed by SPSS 26.0 statistical software (IBM Corp., Group NY). The expert positivity factor (E) is generally expressed in terms of the questionnaire return rate and measures the level of motivation and involvement of experts in the consultation. According to previous studies, the expert motivation factor should be at least 50% or more; above 60% indicates a high level of motivation, and 70% and above indicate a high level of motivation (37). The degree of authority of the experts’ opinions is reflected by the coefficient of the basis of the experts’ judgements on each indicator (Ca) and the coefficient of their familiarity with each indicator (Cs). The authority coefficient (Cr) is equal to the arithmetic mean of the coefficient of judgement basis and the coefficient of familiarity, i.e., Cr=(Ca+Cs)/2. The range of values for Cr was 0-0.95, and the critical value for more credible results was ≥0.7 (36). Coefficients of variation and Kendall’s coefficient of coordination (W-values) are used to indicate the degree of consistency of expert opinion.

## 3 Results

### 3.1 Demand for case management information platforms from breast cancer patients and families

A total of 231 questionnaires were collected, including 189 patients (81.8%) and 42 family members (18.2%), who had an average age of 50.3 years. The vast majority of the respondents in this survey were women (207, 89.6%), and they were married (187,81.0%). The vast majority of patients were in the surgery stage (55, 23.8%) or chemotherapy stage (60, 26.0%). Regarding the progression of the disease, 46.3% of the participants were unaware of it. Specific information can be found in **Appendix 5**. The results of the evaluation of the content of the CM information module based on the theoretical framework of PPV by patients and their families are shown in **Table 1**.

**Table 1:** Content needs and evaluation of the information management platform by breast cancer patients and family members.

Function	Time	Sevices	$\bar{X}\pm S$
**Functional value**	**Pre-admission**	Provide appointment booking service	4.60 ± 0.603
		If not	2.77 ± 1.436
		Push information about the treatment process	4.53 ± 0.631
		If not	2.76 ± 1.381
	**In hospital**	Establishing a health record	4.56 ± 0.662
		If not	2.85 ± 1.415
		Individualised care plans	4.54 ± 0.587
		If not	2.84 ± 1.353
		Tracking Management	4.57 ± 0.577
		If not	2.75 ± 1.398
		Prevention and management of complications	4.54 ± 0.617
		If not	2.74 ± 1.351
		Dietary and lifestyle guidance	4.54 ± 0.609
		If not	2.70 ± 1.352
	**After hospital**	Targeted health education	4.55 ± 0.629
		If not	2.76 ± 1.358
		Management of concomitant symptoms during treatment and rehabilitation	4.54 ± 0.580
		If not	2.78 ± 1.357
		Out of hospital follow up	4.51 ± 0.632
		If not	2.76 ± 1.338
		Health education for carers	4.48 ± 0.684
		If not	2.92 ± 3.104
		Promote online health education knowledge	4.49 ± 0.678
		If not	2.77 ± 3.750
**Emotional value**	**Pre-admission**	Contact the medical team online at any time for a consultation	4.56 ± 0.607
		If not	2.72 ± 1.365
	**In hospital**	Provide a dedicated person (case manager) for long-term follow-up	4.54 ± 0.631
		If not	2.76 ± 1.365
		Regular assessments by case managers	4.56 ± 0.608
		If not	2.74 ± 1.370
		Multiple approaches to psycho-emotional support	4.52 ± 0.617
		If not	2.96 ± 3.506
	**After hospital**	Provide case manager contact details	4.52 ± 0.638
		If not	2.74 ± 1.361
		Patient Exchange Platform	4.55 ± 0.594
		If not	2.79 ± 1.338
		Real-time online consultation	4.52 ± 0.596
		If not	2.74 ± 1.358
**Value of efficiency**	**Pre-admission**	Case managers to book specialist appointments for you	4.52 ± 0.617
		If not	2.75 ± 1.366
		Special Disease Process	4.79 ± 3.370
		If not	2.75 ± 1.370
		Hospital access information support	4.54 ± 0.580
		If not	2.78 ± 1.341
	**In hospital**	Full rehabilitation needs assessment	4.56 ± 0.600
		If not	2.84 ± 1.397
		Information on Venous Access Maintenance Clinics and Community Maintenance Sites	4.42 ± 0.730
		If not	2.79 ± 1.322
		Regional medical referrals	4.42 ± 0.718
		If not	2.77 ± 1.320
	**After hospital**	Nurse visits	4.41 ± 0.697
		If not	2.85 ± 1.312
		Teleconsultation	4.46 ± 0.664
		If not	2.83 ± 1.342
**Social values**	**Pre-admission**	Green channel to medical treatment	4.55 ± 0.601
		If not	2.69 ± 1.372
	**In hospital**	Provide individualised guidance to enhance patients’ ability to manage their own rehabilitation	4.70 ± 2.016
		If not	2.77 ± 1.337
	**After hospital**	Health Education Seminar Live Event	4.51 ± 0.618
		If not	2.78 ± 1.344
		Provide addresses and contact numbers of health care centres and communities in each district and county	4.49 ± 0.652
		If not	2.79 ± 1.332
		Provide contact details for social assistance agencies (e.g. Cancer Relief Foundation)	4.48 ± 0.678
		If not	2.81 ± 1.336

### 3.2 Basic information and positive coefficients for experts

Twenty-two experts in clinical areas, nursing education and nursing management related to BC care were purposively selected from nursing schools and departments of major universities and tertiary hospitals in the southwest region according to predetermined criteria for the selection of experts. In the first round of the study, 22 consultation questionnaires were distributed, and 18 valid questionnaires were returned, for a positive coefficient of 81.8%; in the second round of the study, 18 consultation questionnaires were distributed, and 18 valid questionnaires were returned, for a positive coefficient of 100%. The distribution of the general information of the included experts is shown in **Table 2**.

**Table 2:** Basic information on the 18 experts included in this correspondence.

Items	Number	Percentage (%)
**Title**		
Intermediate	12	66.7
Associate Senior	5	27.8
Senior	1	5.6
**Academic qualifications**		
Bachelor’s degree	10	55.6
Master’s degree	7	38.9
Doctor	1	5.6
**Fields of work**		
Clinical	10	55.6
Education	2	11.1
Management	6	33.3
**Age (years)**		
<30	4	22.2
30∼40	6	33.3
41∼50	6	33.3
>50岁	2	11.1
**Years of work(years)**		
<10	7	38.9
10∼20	5	27.8
21∼30	5	27.8
>30	1	5.6

### 3.3 Expert authority factor

The results show that four experts were very familiar with the indicators, 11 were more familiar and three were generally familiar. In addition, the experts judged each indicator on the basis of **Table 3**. The authority level of the experts’ opinions in this study was 0.87, indicating that the experts were more authoritative.

**Table 3:** Analysis of the basis of judgement of the 18 experts.

Basis of judgement	Degree of impact		
	Great	Medium	Little
Theoretical analysis	12	6	0
Practical experience	12	6	0
Bibliography	7	6	5
Subjective judgement	4	3	11

### 3.4 The degree of coordination of expert opinion

The mean values of the coefficients of variation of the indicators in the 2 rounds of the study ranged from 0.094 to 0.175, and the differences were statistically significant (p< 0.05). The values of the Kendall harmonic coefficients for the various levels are shown in **Table 4**.

**Table 4:** Level of coordination of expert opinion.

Rounds	Levels	Mean value of coefficient of variation	W value	X^2^	df	P
**Round 1**	Tier 1 indicators	0.069	0.178	8.538	3	0.036
	Secondary indicators	0.070	0.084	37.412	28	0.110
**Round 2**	Tier 1 indicators	0.080	0.296	14.186	3	0.003
	Secondary indicators	0.081	0.136	65.499	30	<0.001

W, Kendall cofficient of concordance; X^2^, Chi-square test; df, degree of freedom.

### 3.5 Selection and identification of indicators

Through two rounds of expert consultation, the average importance score for all indicators ranged from 4.50 to 5.00, and the coefficient of variation ranged from 0 to 0.181; items were screened on the basis of an average importance score > 3.50 and a coefficient of variation< 0.25, and no indicators were deleted. However, it was noted that B2.2 Health lectures were a duplicate of A3.2 Health education and therefore, the item was removed. Three new secondary indicators were added: “B2.2 Counselling”, “B3.3 Family support” and “D3.3 Emergency access”. The content of the indicators A3.1 Follow-up tracking and C1.1 Consultation services was revised and adjusted in conjunction with expert opinion. Through the second round of consultation, four primary indicators and 31 secondary indicators were identified, and their mean scores, standard deviations and coefficients of variation are shown in **Table 5**.

**Table 5:** Evaluation of secondary indicators of the Breast Cancer Case Management Information Platform module.

Tier 1 Indicator		Secondary Indicator	Importance score		Coefficient of variation
			$\bar{X}$	S	
**Functional value** **(A)**	**Pre-Admission** **(A1)**	**Consultation services (A1.1)**	4.75	.577	0.121
		**Early screening (A1.2)**	5.00	.000	0
		**Outpatient medical records (A1.3)**	4.88	.500	0.102
	**In hospital** **(A2)**	**Health record (A2.1)**	4.88	.342	0.070
		**Inpatient records (A2.2)**	4.94	.250	0.051
		**Treatment tracking management (A2.3)**	5.00	.000	0
		**Health guidance (A2.4)**	4.88	.342	0.070
	**After hospital** **(A3)**	**Follow-up tracking (A3.1)**	5.00	.000	0
		**Health education (A3.2)**	4.81	.403	0.084
**Emotional value** **(B)**	**Pre-admission(B1)**	**Pre-visit consultation (B1.1)**	4.56	.512	0.112
	**In hospital (B2)**	**Case assessment (B2.1)**	4.94	.250	0.051
		**Psychological counselling (B2.2)**	4.56	.629	0.138
	**After hospital(B3)**	**Recovery monitoring (B3.1)**	4.94	.250	0.051
		**Patients’ homes (B3.2)**	4.81	.403	0.084
		**Family support (B3.3)**	4.88	.342	0.070
**Value of efficiency** **(C)**	**Pre-admission** **(C1)**	**Consultation services(C1.1)**	4.75	.683	0.144
	**In hospital** **(C2)**	**Early warning of positive tests(C2.1)**	4.94	.250	0.051
		**Specialist referrals(C2.2)**	4.69	.602	0.128
		**Multidisciplinary medical teams(C2.3)**	4.75	.447	0.094
	**After hospital** **(C3)**	**Follow-up(C3.1)**	5.00	.000	0
		**Online consultation(C3.2)**	4.81	.403	0.084
		**Network nursing(C3.3)**	4.56	.727	0.159
		**Statistical analysis(C3.4)**	4.88	.342	0.070
**Social values** **(D)**	**Pre-admission** **(D1)**	**Treatment services(D1.1)**	4.81	.403	0.084
	**In hospital** **(D2)**	**Health education(D2.1)**	4.94	.250	0.051
		**Links to resources(D2.2)**	4.75	.447	0.094
		**Graded diagnosis and treatment(D2.3)**	4.63	.719	0.155
	**After hospital** **(D3)**	**Online consultation(D3.1)**	4.94	.250	0.051
		**Patients’ Home(D3.2)**	4.88	.342	0.070
		**First aid channel(D3.3)**	4.50	.816	0.181
		**Satisfaction surveys(D3.4)**	4.81	.403	0.084

### 3.6 Model framework for a case management information platform for breast cancer patients

**Figure 1:**
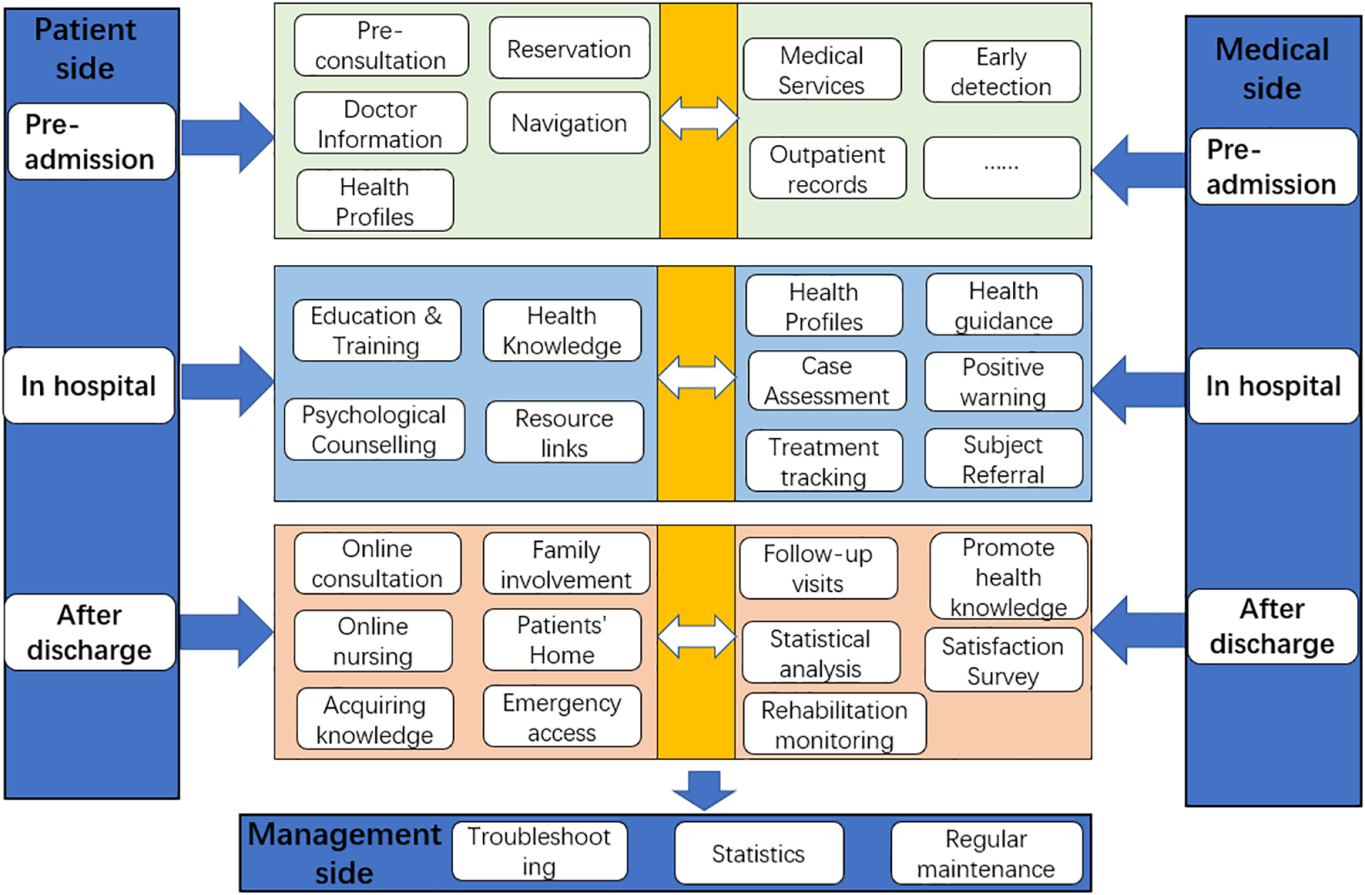
Framework for a case management information platform for breast cancer patients.

The BC-CMIP is divided into a medical side for healthcare professionals and a patient side for patients and family members. The overall framework is shown in **Figure 1**. The medical end of the platform connects to the medical systems of each treatment unit through mobile medical technology, storing the medical examination data, consultation cases, examination results, medication prescriptions and health data uploaded by patients and their families in the platform, forming a complete BC patient’s personal electronic health file and updating the health management records in real time. The case manager and medical staff can access the treatment records of BC patients at any time to understand the consultation results, examination and recovery and implement health management, health guidance, tracking management and business supervision. The management side provides statistics and analysis of health data, identifies alert values when compared with defined criteria, and dynamically monitors the whole process of BC patient management services. The patient side provides an online hospital, health testing, health assessment, expert consultation, patient home and access to health knowledge for patients and family members.

## 4 Discussion

In this study, based on the service needs and evaluation of breast cancer patients and family members on the case management platform, the content module of the whole information platform for breast cancer patients’ case management was initially constructed through two rounds of expert correspondence based on the framework of patients’ perceived value. It is scientific and practical and provides a theoretical basis for the subsequent construction of the case management platform and its clinical application.

As payment models change, with more clinicians and health care entities accepting financial risk for outcomes, health care systems are using digital health to manage their populations and improve access, patient experience, and control costs (38). At the same time, patients’ interest in using technology to manage their health is increasing. Many patients seek information from the internet to learn more about their symptoms, diagnoses, and treatments. An increasing number are also using wearable devices and mobile applications to track their health. In addition, numerous studies now highlight the importance of designing and developing software platforms based on user requirements (38). The first principle of the software platform is to meet the needs of the people who use it because the software platform developed under the guidance of the needs will be more humane and practical and more likely to obtain long-term, stable support from the people who use it and higher application satisfaction. Therefore, this study first investigated the demand for and evaluation of case management information platform services by breast cancer patients and family members, and the results showed that patients and their families have a high opinion of the functional content of the information module for breast cancer patients, with mean scores ranging from 4.41 ± 0.697 to 4.60 ± 0.603, while without the implementation of these items, patient satisfaction scores are all less than 3, i.e., not satisfied. It can be seen that the content of the module of the information platform for case management of breast cancer patients based on the perceived value of patients constructed in this study meets the health needs of patients and is an essential health link.

Studies have shown that case managers spend considerable time recording patient-related information and case management processes (39) and that the key to the effective implementation of breast cancer case management is a well-functioning web-based platform (40). Wang et al. (41) investigated the application of professional case management based on the WeChat platform in BC patients. A total of 149 BC patients were randomly divided into two groups. The difference between the two groups was statistically significant (P<0.05), and the difference between the two groups’ health promotion behaviour scores at 3 months after discharge was statistically significant (P<0.001). Thus, with specialized CM through the information platform, patients can proactively and timely communicate with healthcare professionals and obtain the most direct and reliable professional information. Members of the CM team track, follow up, monitor, intervene and record BC patients’ life and compliance behaviour, forming a feedback system and providing targeted one-to-one guidance, improving the quality of out-of-hospital care, promoting patient self-healing and maintaining the permanence of patient health management.

Bettencourt et al. (42) pointed out in 2008 that service innovation is not a study of how the service is achieved but of how the customer wants to achieve the service. As healthcare is a professional service industry, it is not enough for the healthcare industry to focus on the clinical value of the patient from the doctor’s perspective alone to obtain service innovation; the development of healthcare services also needs to revolve around the patients’ multidimensional perceived value. This study systematically and comprehensively reflects the health needs of patients from prehospital to posthospital based on the four value dimensions of patient-perceived value, namely, functional, efficiency, emotional and social values. In addition, indicators were screened with the help of the Delphi method (32), combining expert opinions with statistical analysis of data, integrating consistency and coordination based on the original opinions of experts, while satisfying the requirement of overall opinion convergence to obtain the optimal solution for group decision-making and indicators with credibility. This combination of subjective and objective methods makes the selection of the indicator system more scientific and appropriate. Therefore, this study constructs a case management information platform for breast cancer patients based on patient-perceived value theory, which is scientific and comprehensive and can meet the needs of patient disease management.

This study has the following limitations. First, there was a lack of information engineers on the expert discussion group for guidance. Second, although we included fill-in-the-blank questions in the needs questionnaire, patients and their family members did not provide much data. This may mean that the needs of patients and some of their family members were not fully included.

## 5 Conclusion

This study takes the PPV of BC patients as the theoretical framework, is demand oriented, and constructs the content of the BC-CMIP module with the help of the Delphi method, which has the scientific and comprehensive ability to meet the health management needs of patients. The information platform can provide patients with convenient access to information and medical and nursing consultation carriers. With the information platform as a carrier, medical and nursing staff can participate in the whole cycle of patients’ disease treatment and rehabilitation, meet patients’ needs for professional guidance, effectively improve breast cancer patients’ self-management ability and improve their survival quality. It can be used as a new mode of case management for BC patients. Due to the limited duration of this study, the next step is to apply this management model to clinical practice and conduct a multicentre, large sample study to further improve the confidence platform for breast cancer case management.

## Data availability statement

The raw data supporting the conclusions of this article will be made available by the authors, without undue reservation.

## Author contributions

XG, supervising the subject throughout, looking for correspondence experts; YL, Extensive literature review, data collection, constructing questionnaires, distributing questionnaires, checking papers. YG, Data collection, constructing questionnaires, data collation, data analysis and collation, writing the thesis and thesis reworking. YL and YG contributed equally to this work and share first authorship. GY, Data collection, methodological guidance. WC, Data collection. All authors contributed to the article and approved the submitted version.

## Funding

Project title: Study on the impact of chemotherapy-induced peripheral neuropathy on the quality of life of breast cancer patients and its countermeasures Item No.:2019MSXM068

## Conflict of interest

The authors declare that the research was conducted in the absence of any commercial or financial relationships that could be construed as a potential conflict of interest.

## Publisher’s note

All claims expressed in this article are solely those of the authors and do not necessarily represent those of their affiliated organizations, or those of the publisher, the editors and the reviewers. Any product that may be evaluated in this article, or claim that may be made by its manufacturer, is not guaranteed or endorsed by the publisher.

## References

[1] Organization WH. World health organization projections of mortality and causes of death . Available at: https://www.Who.int/healthinfo/global_burden_disease/projections/en.

[2] Chen W Zheng R Baade PD Zhang S Zeng H Bray F. Cancer statistics in China, 2015. CA Cancer J Clin (2016) 66(2):115–32. doi: 10.3322/caac.21338 26808342

[3] Feng RM Zong YN Cao SM Xu RH. Current cancer situation in China: good or bad news from the 2018 global cancer statistics. Cancer Commun (Lond). (2019) 39(1):22. doi: 10.1186/s40880-019-0368-6 31030667 PMC6487510

[4] Global Burden of Disease Cancer Collaboration Fitzmaurice C Akinyemiju TF Al Lami FH Alam T Alizadeh-Navaei R. Global, regional, and national cancer incidence, mortality, years of life lost, years lived with disability, and disability-adjusted life-years for 29 cancer groups, 1990 to 2016: A systematic analysis for the global burden of disease study. JAMA Oncol (2018) 4(11):1553–68. doi: 10.1001/jamaoncol.2018.2706 PMC6248091 29860482

[5] Thorat MA Balasubramanian R. Breast cancer prevention in high-risk women. Best Pract Res Clin Obstet Gynaecol. (2020) 65:18–31. doi: 10.1016/j.bpobgyn.2019.11.006 31862315

[6] Paluch-Shimon S Pagani O Partridge AH Abulkhair O Cardoso MJ Dent RA. ESO ESMO 3rd international consensus guidelines for breast cancer in young women (BCY3). Breast (2017) 35:203–17. doi: 10.1016/j.breast.2017.07.017 28822332

[7] Boundouki G Wilson R Duxbury P Henderson J Ballance L Wray J. Patient and public priorities for breast cancer research: a qualitative study in the UK. BMJ Open (2021) 11(1):e036072. doi: 10.1136/bmjopen-2019-036072 PMC7849895 33514570

[8] Kim S Han J Lee MY Jang MK. The experience of cancer-related fatigue, exercise and exercise adherence among women breast cancer survivors: Insights from focus group interviews. J Clin Nurs. (2020) 29(5-6):758–69. doi: 10.1111/jocn.15114 31769562

[9] Liu L Wu Y Cong W Hu M Li X Zhou C. Experience of women with breast cancer undergoing chemotherapy: a systematic review of qualitative research. Qual Life Res (2021) 30(5):1249–65. doi: 10.1007/s11136-020-02754-5 33459972

[10] Ao L. The application of self-management mode based on empowerment theory in patients with postoperative chemotherapy for breast cancer. Shanxi Med Univ (2019).

[11] Li JH Li XY Tan Y Wei T. Application of the multidisciplinary-team management model in patients with breast cancer’s chemotherapy-induced nausea and vomiting. Chin Nurs Manage (2018) 18(03):367–72. doi: 10.3969/j.issn.1672-1756.2018.03.018

[12] Sun W Chen Y. Application of 5S health education management model in health education for breast cancer patients. J Nurs Administration (2018) 18(05):373–376+380. doi: 10.3969/j.issn.1671-315x.2018.05.018

[13] Jary J Franklin L. The role of the specialist nurse in breast cancer. Prof Nurse. (1996) 11(10):664–5. doi: 10.1007/978-1-4419-6076-4_3 8718373

[14] Chen YZ Zhou YZ. The case managerment experience in Taipei veterans general hospital. Chin Hosp Manag (2010) 10(3):21–2.

[15] Zhang LL. The development and future of tumor case managers. J Cancer Care (2010) 10(1):19.

[16] Zhang LL. The current situation and prospect of case management in Taiwan//Training courses for cancer prevention professionals-role and function of tumor case manager. Taichung: Affiliated Hosp China Med Univ (2010) 70–75.

[17] Giuliano KK Poirier CE. Nursing case management: critical pathways to desirable outcomes. Nurs Manage (1991) 22(3):52–5. doi: 10.1097/00006247-199103000-00015 1900600

[18] Dieterich M Irving CB Bergman H Khokhar MA Park B Marshall M. Intensive case management for severe mental illness. Cochrane Database Syst Rev (2017) 1(1):CD007906. doi: 10.1002/14651858.CD007906.pub3 28067944 PMC6472672

[19] Reilly S Miranda-Castillo C Malouf R Hoe J Toot S Challis D. Case management approaches to home support for people with dementia. Cochrane Database Syst Rev (2015) 1(1):CD008345. doi: 10.1002/14651858.CD008345.pub2 25560977 PMC6823260

[20] Aubin M Giguère A Martin M Verreault R Fitch MI Kazanjian A. Interventions to improve continuity of care in the follow-up of patients with cancer. Cochrane Database Syst Rev (2012) 7:CD007672. doi: 10.1002/14651858.CD007672.pub2 PMC11608820 22786508

[21] Peng CE Wang WH Li Z Chen YY Chen XB Cai X. Influence of case management model on quality of life and psychosocial adaptation of patients with breast cancer. Nurs Res (2015) 10(29):3541–5. doi: 10.3969/j.issn.1009-6493.2015.28.029

[22] Gan L Lin YH Yang R. The development and present situation of nursing in Taiwan. Nurs Res (2014) 12(28):4491–3. doi: 10.3969/j.issn.10096493.2014.36.004

[23] Chen YC Chang YJ Tsou YC Chen MC Pai YC. Effectiveness of nurse case management compared with usual care in cancer patients at a single medical center in Taiwan: a quasi-experimental study. BMC Health Serv Res (2013) 13:202. doi: 10.1186/1472-6963-13-202 23725552 PMC3673875

[24] Wulff CN Vedsted P Søndergaard J. A randomized controlled trial of hospital-based case management in cancer care: a general practitioner perspective. Fam Pract (2013) 30(1):5–13. doi: 10.1093/fampra/cms050 22952209

[25] Hubbard G Gray NM Ayansina D Evans JM Kyle RG. Case management vocational rehabilitation for women with breast cancer after surgery: a feasibility study incorporating a pilot randomised controlled trial. Trials. (2013) 14:175. doi: 10.1186/1745-6215-14-175 23768153 PMC3698180

[26] Lantz PM Keeton K Romano L Degroff A. Case management in public health screening programs: the experience of the national breast and cervical cancer early detection program. J Public Health Manag Pract (2004) 10(6):545–55. doi: 10.1097/00124784-200411000-00012 15643379

[27] Wang H Wang H. The factors influencing insured people seeking remote medical treatment– the suggestion for orderly seeking medical treatment. Chin Heal Insur. (2014) 7:122–7. doi: 10.369/j.issn.1674-3830.2014.7.4

[28] Gallarza M Gil S. Value dimensions perceived value, satisfaction and loyalty: an investigation of university students. Tour Manag (2004) 27(03):437–52. doi: 10.1016/j.tourman.2004.12.002

[29] Zhang X Sleeboom-Faulkner M. Tensions between medical professionals and patients in mainland China. Camb Q Healthc Ethics. (2011) 20(3):458–65. doi: 10.1017/S0963180111000144 21676333

[30] Qian H Lu W Zhang D. Empirical investigation on the characteristics and perceived value of patients in medical treatment seeking: In-depth research in zhejiang province of China. BioMed Res Int (2021) 2021:5245041. doi: 10.1155/2021/5245041 34977240 PMC8720011

[31] Hu R Chen HF Xu WG. A case study on the formation mechanism of the patients perceived value in mobile medical system. Manage Review. (2017) 29(03):261–72. doi: 10.3969/j.issn.1672-0334.2018.03.007

[32] McPherson S Reese C Wendler MC. Methodology update: Delphi studies. Nurs Res (2018) 67(5):404–10. doi: 10.1097/NNR.0000000000000297 30052591

[33] Keeney S Hasson F McKenna H. The Delphi technique in nursing and health research. West Sussex, UK: Wiley-Blackwell (2011).

[34] Stitt-Gohdes WL Crews R. The Delphi technique: A research strategy for career and technical education. CTE J (2004) 20:55–67. doi: 10.21061/jcte

[35] McMillan SS King M Tully MP. How to use the nominal group and Delphi techniques. Int J Clin Pharm (2016) 38(3):655–62. doi: 10.1007/s11096-016-0257-x PMC4909789 26846316

[36] Zeng G. Modern epidemiological methods and applications. China: Beijing Medical University, China Union Medical University Press (1994).

[37] Macdonald EB Ritchie KA Murray KJ Gilmour WH. Requirements for occupational medicine training in Europe: a Delphi study. Occup Environ Med (2000) 57(2):98–105. doi: 10.1136/oem.57.2.98 10711277 PMC1739906

[38] Agboola SO Bates DW Kvedar JC. Digital health and patient safety. JAMA. (2016) 315(16):1697–8. doi: 10.1001/jama.2016.2402 27115372

[39] Armold S. Case management: An overview for nurses. Nursing. (2019) 49(9):43–5. doi: 10.1097/01.NURSE.0000577708.49429.83 31436722

[40] Buescher C Thorenz A Grochocka A Koch U Watzke B. Die Case-Management-basierte Betreuung von Brustkrebspatientinnen: Ergebnisse einer Befragung beteiligter ärztlicher und nichtärztlicher Netzwerkpartner [A case management programme for women with breast cancer: Results of a written survey of participating medical and non-medical, networking-partners. Gesundheitswesen (2011) 73(12):815–22. doi: 10.1055/s-0030-1262863 21110297

[41] Wang ZJ Lin ZJ Ma HX. Application effect of specialized case management based on WeChat mobile platform in extended service of breast cancer patients. Nurs Res (2019) 33(3):524–7. doi: 10.12102/j.issn.1009-6493.2019.03.043

[42] Bettencourt LA Ulwick AW. The customer-centered innovation map. Harv Bus Rev (2008) 86(5):109–14, 130. doi: 10.12102/j.issn.1009-6493.2 18543812

