# Supplementary material for: Development of a breast cancer case management information platform (BC-CMIP) module based on patient-perceived value
Source: Front Oncol. 2022 Nov 28;12:1034171. doi: 10.3389/fonc.2022.1034171 (PMC9742556; doi:10.3389/fonc.2022.1034171)
Supplement: Supplementary file 1 [file DataSheet_1.pdf]

Appendix 1 A survey of demand for full case management services based on perceived value

No.:____________

Informed consent form for a full case management service needs survey based on perceived value

Dear Participants,

We are the medical staff of the Breast Disease Case Management Team at the Second Hospital of Chongqing Medical University. Breast cancer is now a global public health problem. In order to manage the disease and improve your quality of life, our team is conducting this anonymous survey to understand your needs for a full range of management services for the disease and to provide a theoretical basis for the development of a targeted full range of management programmes. This survey is for academic research only and your answers will be treated in strict confidence and no personal information will be disclosed. It will take about 3-5 minutes to complete this questionnaire, so please answer all the questions below according to your real situation, the information you provide will be of great help to the implementation of this study.

Breast Cancer Case Management Team
The Second Hospital of Chongqing Medical University

I have been briefed by the investigator on the purpose and process of this study, understand that there will be no harm to my mind, body, treatment or care, and I agree to participate in the data collection process on the subject.

Name:_____________

Date:______________

I. Basic information

1. Age(years):_______________
2. Gender: ①Male ②Female
3. Your status: ① patient ② family member (please specify your relationship to the patient)
4. What stage of treatment are you (or your family) currently in?

   ①Confirmation ②Surgery ③Chemotherapy ④Radiotherapy ⑤Targeted ⑥Endocrine therapy ⑦Recovery ⑧Other:__________
5. Your (or your family member's) current stage of disease:

   ①Stage I ② Stage II ③ Stage III ④ Stage IV ⑤ Unknown ⑥ Other:_________
6. Ethnicity: ① Han ② Minority (please specify):_______________
7. Marital status: ① unmarried ② married ③ divorced ④ widowed ⑤ separated
8. Religion: ①None ②Buddhism ③Christianity ④Other:______________
9. Education level:

   ① Junior high school and below ② High school/junior college ③ College/bachelor's degree ④ Master's degree and above
10. Occupation:

    ①Civil servant ②Teacher ③Medical personnel ④Other institution personnel ⑤Worker ⑥Enterprise personnel ⑦Other:________
11. Average monthly gross income (RMB):

    ①3000 and below ②3001-5000 ③5001-8000 ④8000 and above

12.Medical payments:

① New Agricultural Cooperative ② Urban residents' medical insurance ③ Urban workers' medical insurance ④ Commercial insurance ⑤ None

13.Place of permanent residence: ① rural areas ② towns ③ cities

II. Patient and Family Member Service Needs Survey

Listed below are some questions to understand your service needs, please tick the closest of the 5 answers after each question to your actual situation

**Contents**		**Very satisfactory**	**Relatively satisfactory**	**Uncertain**	**Relatively unsatisfactory**	**Very unsatisfactory**
1.Provide appointment booking service for you(or your family members)	Provide this service					
	This service is not available					
2.Establishing a health record for you(or your family members)	Provide this service					
	This service is not available					
3.A full rehabilitation needs assessment for you(or your family members)	Provide this service					
	This service is not available					
4.Individualised care planning for you (or your family members)	Provide this service					
	This service is not available					
5.Tracking management for you (or your family members) during treatment	Provide this service					
	This service is not available					

6.Health education tailored to the stage of treatment you (or your family members) are in	Provide this service					
	This service is not available					
7.Prevention and management of complications of treatment for you (or your family members)	Provide this service					
	This service is not available					
8.Provide symptom management for you (or your family members) during treatment and rehabilitation	Provide this service					
	This service is not available					
9.Out-of-hospital follow-up for you (or your family members)	Provide this service					
	This service is not available					
10.Guidance for you (or your family members) on the use of rehabilitation products	Provide this service					
	This service is not available					
11.Dietary and lifestyle guidance for you (or your family members)	Provide this service					
	This service is not available					
12.Carer health education for you (or your family members)	Provide this service					
	This service is not available					
13.Online health education for you (or your family members)	Provide this service					
	This service is not available					
14.Live online advice for you (or your family members)	Provide this service					
	This service is not available					
15.Review reminders for you (or your family members)	Provide this service					
	This service is not available					
16.Green access for you (or your family members)	Provide this service					
	This service is not available					

17.Push information about the treatment process for you(or your family members)	Provide this service					
	This service is not available					
18.The case manager will make an appointment for you (or your family members) to see a specialist according to your medical needs	Provide this service					
	This service is not available					
19.You (or your family members) can contact the medical team online for a consultation while you are out of hospital	Provide this service					
	This service is not available					
20.Nurse home care service for you (or your family members)	Provide this service					
	This service is not available					
21.Teleconsultation for you (or your family members)	Provide this service					
	This service is not available					
22.Regional medical referrals for you (or your family members)	Provide this service					
	This service is not available					
23.Provide you (or your family members) with the contact details of a case manager so that the medical team can be contacted at any time	Provide this service					
	This service is not available					
24.Provide you (or your family members) with a dedicated person (case manager) for long-term follow-up and close contact with your medical team	Provide this service					
	This service is not available					
25.Regular assessment of your (or your family members) recovery by a case manager	Provide this service					
	This service is not available					
26.A wide range of psycho-emotional support options for you (or your family members)	Provide this service					
	This service is not available					

27.Live health education talks for you (or your family members)	Provide this service					
	This service is not available					
28.To provide a platform for you (or your family members) to communicate with your patients and share knowledge about recovery	Provide this service					
	This service is not available					
29.Individualised guidance for you (or your family members) to enhance your (or your family's) self-management of your rehabilitation	Provide this service					
	This service is not available					
30.Support you (or your family members) with information on hospital visits	Provide this service					
	This service is not available					
31.Provide you (or your family members) with addresses and contact numbers of health care centres and communities in each district and county	Provide this service					
	This service is not available					
32.Provides you(or your family members) with information on venous access maintenance clinics and community maintenance sites	Provide this service					
	This service is not available					
33.Special illness process for you (or your family members)	Provide this service					
	This service is not available					
34.Provide you (or your family members) with contact details for social assistance organisations (e.g. Cancer Support Foundation)	Provide this service					
	This service is not available					
35.Regular patient networking meetings to provide you (or your family members) with peer encouragement and support	Provide this service					
	This service is not available					

**For other service requests, please fill in the space to the right**	

III. Would you be willing to pay if the hospital offered you (or your family) full case management services (i.e. the above service entry)?

①Yes ②No

IV. Would you recommend the hospital case management service to a patient close to you?

①Yes ②No

Appendix 2 Expert Letter Form (Round 1)

Building a Breast Cancer Case Management Information Platform Based on Patient Perceived Value Theory

Dear Experts,

We are the breast cancer case management team at the Second Hospital of Chongqing Medical University and are conducting a study on the construction of an information platform for breast cancer case management. Patient perceived value refers to the overall evaluation of the utility of health care services by weighing the benefits and losses that patients can perceive in the process of receiving health care services, and is an extension of the customer perceived value theory in the field of health care services. Based on this theory, the system is a comprehensive case management information platform that integrates pre-hospital screening, in-hospital comprehensive management and post-hospital follow-up of breast cancer patients, which can promote the efficiency and service capacity of hospital case management. After extensive literature research and expert panel discussions, the project team has initially developed 4 level 1 entries and 29 level 2 entries.

We sincerely thank you for your support and assistance! Due to the needs of the project, we would be grateful if you could send us your feedback within **TWO WEEKS**. We wish you good health and success in your work!

Breast Cancer Case Management Team
The Second Hospital of Chongqing Medical University

Part I:Evaluation Indicator System Consultation Form

I. Tier 1 Indicator Consultation Form

Items	Contents	Importance of indicators					Comments
		Very important (5 points)	Relatively important (4 points)	Generally important (3 points)	Not very important (2 points)	Very unimportant (1 point)	
**Functional value(A)**	Convenient features of the information platform for patient access and medical staff consultation and treatment						
**Emotional value(B)**	Information platforms provide emotional support and humanistic care for patients						
**Value of efficiency(C)**	The value of efficiency and effectiveness of treatment for patients coming to the hospital						
**Social values(D)**	Patients assign their own value as a social being, and illness and treatment can affect the social value of patients						
**If there are new indicators, please write the name of the indicator here and answer the options in the right column.**							

II. Secondary Indicator Consultation Form

Tier 1		Secondary	Contents	Importance of indicators	Comments

Indicator		Indicator		Very important (5 points)	Relatively important (4 points)	Generally important (3 points)	Not very important (2 points)	Very unimportant (1 point)	
**Functional value (A)**	**Pre-admission (A1)**	**Consultation services(A1.1)**	Including appointment booking, consultation navigation, consultation reminders						
		**Early screening (A1.2)**	Patients are alerted by the system when their mammogram reports BIRADS of category 4, 5 or 6, and case managers follow up and record in a timely manner to ensure that patients are perfected for further treatment						
		**Outpatient medical records (A1.3)**	Mainly include outpatient visits, physical examinations, test reports, medical information, medication prescriptions and so on. Members of the case management team can access the records and the patient side can also view the health records.						
	**In hospital (A2)**	**Health record (A2.1)**	Contains history of previous illness, history of current illness, case assessment, examination reports and follow-up records						
		**Inpatient records(A2.2)**	Access to the HIS system, accessible by the case management team and printable by patients as discharge summaries						
		**Treatment tracking management(**	Treatment stage progress display for case management team to view treatment plan and implementation; adverse reaction tracking:						

		**A2.3)**	patient completes adverse reaction self-assessment record form; case manager visits regularly and records interventions and effects						
		**Health guidance (A2.4)**	Regularly organize various forms of health talks (online + offline) in the hospital, provide key points of home care at different stages of breast cancer treatment and provide comprehensive and systematic information to patients; provide guidance on the use of rehabilitation products such as breast prostheses and wigs						
	**After hospital (A3)**	**Follow-up tracking (A3.1)**	Regular follow-up and assessment of patients' treatment and recovery, and provision of targeted interventions and advice to facilitate early recovery						
		**Health education (A3.2)**	According to the different stages of breast cancer treatment, the system automatically pushes the corresponding knowledge and education content to the patients, providing comprehensive and systematic information to patients and their families, and the case manager can also push it separately						
**Emotional value (B)**	**Pre-Admission (B1)**	**Pre-visit consultation (B1.1)**	Pre-visit consultation reduces patient blindness and anxiety						

	**In hospital (B2)**	**Case assessment (B2.1)**	Case managers establish close contact with patients during patient tracking and management, optimise treatment processes and services, promptly address patient symptoms and barriers to access, improve treatment efficiency and patient perceived value						
		**Health lectures(B2.2)**	Regular announcements of health lectures (online + offline) to promote knowledge sharing						
	**After hospital (B3)**	**Recovery monitoring (B3.1)**	A case manager follows up on a long-term basis to monitor recovery, and patients and the medical team can get in touch at any time to facilitate treatment and consultation and relieve patient anxiety						
		**Patients' homes**	Provide patients with social support: share life experiences in health, exercise, diet and living, etc. Patients' groups provide spiritual support to relieve anxiety and depression and enhance their sense of well-being						
**Value of efficiency (C)**	**Pre-Admission (C1)**	**Consultation services (C1.1)**	Case managers make outpatient appointments for patients, reducing patient appointment times, shortening patient access pathways, improving access efficiency and providing targeted specialties to avoid patients bouncing between multiple specialties, which is time						

			consuming.						
	**In hospital (C2)**	**Early warning of positive tests (C2.1)**	HIS-based tracking and early warning of positive tests improves the accuracy of the case manager's work; patients get more time to communicate with the doctor and patient; reduces treatment complications, promotes doctor-patient communication and improves the efficiency of treatment						
		**Specialist referrals (C2.2)**	Case managers refer patients to specialist clinics such as psychology, rehabilitation and sleep based on patient assessment results to streamline the consultation process						
		**Multidisciplinary medical teams (2.3)**	Provide a space for medical teams to communicate and facilitate discussion of patients' conditions and multidisciplinary consultations						
	**After hospital (C3)**	**Follow-up (C3.1)**	Long-term follow-up by case managers after hospitalisation to improve patients' compliance with review and medication, reduce the recurrence rate of disease, promote early diagnosis and treatment of recurrent patients, and prolong patients' survival						
		**Online consultation (C3.2)**	Provide real-time online answers to patients' home care questions to reduce the number of non-essential hospital visits and improve the efficiency of treatment						

		**Network nursing (C3.3)**	Case managers refer contracted network nurses based on patient follow-up assessments: e.g. wound stoma, PICC/PORT, etc.; network nurse care records and feedback to case management team; patient feedback						
		**Statistical analysis (C3.4)**	Analyse case manager workload and disease monitoring indicators through the information platform to promote efficiency and quality improvement of case management services						
**Social values (D)**	**Pre-admission (D1)**	**Treatment services (D1.1)**	Online booking saves time and effort for patients to visit the clinic						
	**In hospital (D2)**	**Health education (D2.1)**	Case managers provide individualised guidance according to the patient's cognitive level to enhance the patient's self-management skills, promote early recovery and reintegration into society, and reduce the burden on the family and society						
		**Links to resources (D2.2)**	Provide addresses and contact numbers of medical insurance centres and communities in each district and county; provide information on intravenous access maintenance clinics and community maintenance sites; provide special disease processing procedures; provide contact details of various cancer relief foundations						

		**Graded diagnosis and treatment (D2.3)**	Tthe patient side can print referral referrals; provide remote consultation (medical and nursing) to promote sharing of medical resources						
	**After hospital (D3)**	**Online consultation (D3.1)**	Online consultation increases patients' understanding of doctors and diseases, increases doctor-patient trust and promotes doctor-patient harmony						
		**Patients' Home (D3.2)**	Regularly publish the activities of the Patients' Association, showcase recovery cases, share recovery experience and gain peer support						
		**Satisfaction surveys (D3.3)**	Continuous quality improvement through case management satisfaction surveys and patient feedback						
**If there are new indicators, please write the name of the indicator here and answer the options in the right column.**									

Part II, Basic information about the expert

1. Name: ___________ 2.Age(years)：______________ 3. Title：___________________

4.Educational qualifications：____________________ 5.Years of work：__________________

6.How familiar you are with the survey content？

Very familiar ☐ More familiar ☐ Generally familiar ☐ Not familiar ☐ Very unfamiliar ☐

7.The basis for your judgement of the above indicators and the extent of their impact.

**Basis of judgement**	**Degree of impact**		
	**Great**	**Medium**	**Little**
Theoretical analysis			
Practical experience			
Bibliography			
Subjective judgement			

Appendix 3 Table quantifying the basis of judgement and the extent of its impact

Basis of judgement	Degree of impact		
	Great	Medium	Little
Theoretical analysis	0.5	0.4	0.3
Practical experience	0.3	0.2	0.1
Bibliography	0.1	0.1	0.1
Subjective judgement	0.1	0.1	0.1

Appendix 4   Expert Letter Form (Round 2)

Building a Breast Cancer Case Management Information Platform Based on Patient Perceived Value Theory

Dear Experts.

We are the breast cancer case management team at the Second Hospital of Chongqing Medical University and are conducting a study on the construction of an information platform for breast cancer case management. Patient perceived value refers to the overall evaluation of the utility of health care services by weighing the benefits and losses that patients can perceive in the process of receiving health care services, and is an extension of the customer perceived value theory in the field of health care services. Based on this theory, the system is a comprehensive case management information platform that integrates pre-hospital screening, in-hospital comprehensive management and post-hospital follow-up of breast cancer patients, which can promote the efficiency and service capacity of hospital case management. After extensive literature research and the first round of expert consultation, the project team has revised the entries into 4 level 1 entries and 31 level 2 entries.

We sincerely thank you for your support and assistance! Due to the needs of the project, we would be grateful if you could send us your feedback within **ONE WEEK**. We wish you good health and success in your work!

Breast Cancer Case Management Team
The Second Hospital of Chongqing Medical University

Part I:Evaluation Indicator System Consultation Form

I. Tier 1 Indicator Consultation Form

Items	Contents	Importance of indicators					Comments
		Very important (5 points)	Relatively important (4 points)	Generally important (3 points)	Not very important (2 points)	Very unimportant (1 point)	
**Functional value(A)**	Convenient features of the information platform for patient access and medical staff consultation and treatment						
**Emotional value(B)**	Information platforms provide emotional support and humanistic care for patients						
**Value of efficiency(C)**	The value of efficiency and effectiveness of treatment for patients coming to the hospital						
**Social values(D)**	Patients assign their own value as a social being, and illness and treatment can affect the social value of patients						
**If there are new indicators, please write the name of the indicator here and answer the options in the right column.**							

II. Secondary Indicator Consultation Form

Tier 1 Indicator		Secondary Indicator	Contents	Importance of indicators					Comments
				Very important (5 points)	Relatively important (4 points)	Generally important (3 points)	Not very important (2 points)	Very unimportant (1 point)	
**Functional value (A)**	**Pre-Admission (A1)**	**Consultation services (A1.1)**	Including appointment booking, consultation navigation, consultation reminders						
		**Early screening (A1.2)**	Patients are alerted by the system when their mammogram reports BIRADS of category 4, 5 or 6, and case managers follow up and record in a timely manner to ensure that patients are perfected for further treatment						
		**Outpatient medical records (A1.3)**	Mainly include outpatient visits, physical examinations, test reports, medical information, medication prescriptions and so on. Members of the case management team can access the records and the patient side can also view the health records.						
	**In hospital (A2)**	**Health record (A2.1)**	Contains history of previous illness, history of current illness, case assessment, examination reports and follow-up records						
		**Inpatient records (A2.2)**	Access to the HIS system, accessible by the case management team and printable by patients as discharge summaries						

		**Treatment tracking management (A2.3)**	Treatment stage progress display for case management team to view treatment plan and implementation; adverse reaction tracking: patient completes adverse reaction self-assessment record form; case manager visits regularly and records interventions and effects						
		**Health guidance (A2.4)**	Regularly organize various forms of health talks (online + offline) in the hospital, provide key points of home care at different stages of breast cancer treatment and provide comprehensive and systematic information to patients; provide guidance on the use of rehabilitation products such as breast prostheses and wigs						
	**After hospital (A3)**	**Follow-up tracking (A3.1)**	The system can automatically provide early warning reminders based on monitoring data, and the case manager will give specialist guidance based on monitoring data and health records, and adjust intervention strategies in a timely manner based on feedback information						
		**Health education (A3.2)**	According to the different stages of breast cancer treatment, the system automatically pushes the corresponding knowledge and education content to the patients, providing comprehensive and systematic information to patients and their families, and the case						

			manager can also push it separately						
**Emotional value (B)**	**Pre-Admission(B1)**	**Pre-visit consultation (B1.1)**	Pre-visit consultation reduces patient blindness and anxiety						
	**In hospital (B2)**	**Case assessment (B2.1)**	Case managers establish close contact with patients during patient tracking and management, optimise treatment processes and services, promptly address patient symptoms and barriers to access, improve treatment efficiency and patient perceived value						
		**Psychological counselling (B2.2)**	Establish a psychological counselling room, pay attention to the psychological state of patients and encourage them to talk about their bad emotions; and open a network channel to facilitate patients' consultation						
	**After hospital (B3)**	**Recovery monitoring (B3.1)**	A case manager follows up on a long-term basis to monitor recovery, and patients and the medical team can get in touch at any time to facilitate treatment and consultation and relieve patient anxiety						
		**Patients' homes (B3.2)**	Provide patients with social support: share life experiences in health, exercise, diet and living, etc. Patients' groups provide spiritual support to relieve anxiety and depression and enhance their sense of well-being						

		**Family support** **(B3.3)**	Contact patients' families through online channels to follow up on patients' psychological status and treatment to provide a basis for their follow-up treatment						
**Value of efficiency** **(C)**	**Pre-Admission** **(C1)**	**Consultation services** **(C1.1)**	Case managers make outpatient appointments for patients, or push outpatient and doctor information to the patient side to reduce patient appointment time, shorten the patient's path to consultation, improve the efficiency of seeing a doctor, and provide targeted specialties to avoid patients bouncing around between multiple specialties, which is time-consuming						
	**In hospital** **(C2)**	**Early warning of positive tests** **(C2.1)**	HIS-based tracking and early warning of positive tests improves the accuracy of the case manager's work; patients get more time to communicate with the doctor and patient; reduces treatment complications, promotes doctor-patient communication and improves the efficiency of treatment						
		**Specialist referrals** **(C2.2)**	Case managers refer patients to specialist clinics such as psychology, rehabilitation and sleep based on patient assessment results to streamline the consultation process						
		**Multidisciplinary medical teams**	Provide a space for medical teams to communicate and facilitate discussion of patients' conditions and multidisciplinary						

		(C2.3)	consultations						
	**After hospital** (C3)	**Follow-up** (C3.1)	Long-term follow-up by case managers after hospitalisation to improve patients' compliance with review and medication, reduce the recurrence rate of disease, promote early diagnosis and treatment of recurrent patients, and prolong patients' survival						
		**Online consultation** (C3.2)	Provide real-time online answers to patients' home care questions to reduce the number of non-essential hospital visits and improve the efficiency of treatment						
		**Network nursing** (C3.3)	Case managers refer contracted network nurses based on patient follow-up assessments: e.g. wound stoma, PICC/PORT, etc.; network nurse care records and feedback to case management team; patient feedback						
		**Statistical analysis** (C3.4)	Analyse case manager workload and disease monitoring indicators through the information platform to promote efficiency and quality improvement of case management services						
**Social values** (D)	**Pre-Admission** (D1)	**Treatment services** (D1.1)	Online booking saves time and effort for patients to visit the clinic						
	**In hospital**	**Health education**	Case managers provide individualised guidance according to the patient's cognitive level to						

	**(D2)**	**(D2.1)**	enhance the patient's self-management skills, promote early recovery and reintegration into society, and reduce the burden on the family and society						
		**Links to resources (D2.2)**	provide addresses and contact numbers of medical insurance centres and communities in each district and county; provide information on intravenous access maintenance clinics and community maintenance sites; provide special disease processing procedures; provide contact details of various cancer relief foundations						
		**Graded diagnosis and treatment (D2.3)**	the patient side can print referral referrals; provide remote consultation (medical and nursing) to promote sharing of medical resources						
	**After hospital (D3)**	**Online consultation (D3.1)**	Online consultation increases patients' understanding of doctors and diseases, increases doctor-patient trust and promotes doctor-patient harmony						
		**Patients' Home (D3.2)**	Regularly publish the activities of the Patients' Association, showcase recovery cases, share recovery experience and gain peer support						
		**First aid channel (D3.3)**	set the "one key to call for help" button, the platform can automatically locate and directly call the 120 emergency centre, after the						

			emergency centre understands the patient's situation, it can dispatch the emergency medical personnel of the hospital near the patient to receive treatment and give the patient the corresponding emergency treatment; the platform can simultaneously notify the patient's family and community medical personnel by station messages						
		**Satisfaction surveys (D3.4)**	Continuous quality improvement through case management satisfaction surveys and patient feedback						
**If there are new indicators, please write the name of the indicator here and answer the options in the right column.**									

Part II, Basic information about the expert

2. Name: ___________ 2.Age(years)：______________ 3. Title：___________________

4.Educational qualifications：____________________ 5.Years of work：__________________

6.How familiar you are with the survey content？

Very familiar ☐ More familiar ☐ Generally familiar ☐ Not familiar ☐ Very unfamiliar ☐

7.The basis for your judgement of the above indicators and the extent of their impact.

**Basis of judgement**	**Degree of impact**		
	**Great**	**Medium**	**Little**
Theoretical analysis			
Practical experience			
Bibliography			
Subjective judgement			

Appendix 5 Demographic and professional information of the participants

Characteristics	Option	Answer (n)	Frequency (%)
Gender	Male	24	10.4
	Female	207	89.6
Status	Patients	189	81.8
	Family members	42	18.2
Age(years)	18~30	14	6.1
	31~40	48	20.8
	41~50	78	33.8
	51~60	54	23.4
	61~70	30	13.0
	≥71	7	3.0
Education	Junior high school and below	82	35.5
	High school/junior college	63	27.3
	College/Bachelor's degree	82	35.5
	Master’s degree and above	4	1.7
Marital status	Unmarried	9	3.9
	Married	187	81.0
	Divorced	26	11.3
	Widowed	9	3.9
	Separated	0	0
Ethnicity	Han	210	90.9
	Minority	21	9.1
Treatment stages	Confirmation	31	13.4
	Surgery	55	23.8
	Chemotherapy	60	26.0
	Radiotherapy	1	.4
	Targeted	11	4.8
	Endocrine therapy	34	14.7
	Recovery	32	13.9
	Other	7	3.0
Disease stages	Stage I	41	17.7
	Stage II	49	21.2
	Stage III	16	6.9
	Stage IV	8	3.5
	Unknown	107	46.3
	Other	10	4.3
Religion:	None	216	93.5

	Buddhism	11	4.8
	Christianity	3	1.3
	Other	1	.4
Occupation	Civil servant	9	3.9
	Teacher	18	7.8
	Medical personnel	7	3.0
	Other institution	12	5.2
	Personnel	0	0
	Worker	18	7.8
	Enterprise	45	19.5
	Other	122	52.8
Average monthly gross income (RMB)	3000 and below	102	44.2
	3001-5000	78	33.8
	5001-8000	34	14.7
	8000 and above	17	7.4
Place of permanent residence	Rural areas	33	14.3
	Towns	72	31.2
	Cities	126	54.5
